# Impact of nanochitosan incorporation on the performance of resin-modified glass ionomer luting cement: a comprehensive in vitro study

**DOI:** 10.1186/s12903-025-07546-2

**Published:** 2026-01-12

**Authors:** Mostafa A. Abdelshafi, Nesma Elgohary, Ahmed Shams

**Affiliations:** 1 Dental Biomaterials Department, Faculty of Dentistry, Mansoura University, Mansoura, Egypt; 2 Fixed Prosthodontics Department, Faculty of Dentistry, Mansoura University, Mansoura, Egypt; 3 Prosthodontics Department, Dental Biomaterials Division, Faculty of Oral and Dental Medicine, Alsalam University, Tanta, Egypt

**Keywords:** Nanochitosan, RMGI, Film thickness, Solubility

## Abstract

**Background:**

This study aimed to evaluate the effect of incorporating different concentrations of nanochitosan (NCH) into Fuji Plus resin-modified glass ionomer luting cement (RMGIC; GC Corporation, Tokyo, Japan) on shear bond strength, film thickness, solubility, water sorption, and antibacterial activity.

**Methods:**

The liquid component of fuji Plus RMGIC was modified by incorporating NCH at 0% (control), 5%, 10%, and 20% (v/v). A total of ten specimens were prepared per group for each test. Shear bond strength (SBS) was assessed using human mid-coronal dentine after thermocycling. Film thickness was measured in accordance with ISO 4049 and ANSI/ADA guidelines. Water sorption and solubility were assessed after 28 days in artificial saliva. Antibacterial activity against *Streptococcus mutans* was determined via the agar disc diffusion method. Data were analyzed using one-way ANOVA followed by Tukey’s post hoc test (α = 0.05).

**Results:**

NCH incorporation significantly influenced the tested properties of RMGIC. Bond strength remained unaffected at 5% and 10% but significantly decreased at 20% (*p* ≤ 0.05). Film thickness increased only at 20% (*p* ≤ 0.05). Water sorption showed a concentration-dependent increase, while solubility significantly decreased with increasing NCH content (*p* < 0.0001). Antibacterial activity against *Streptococcus mutans* improved significantly with higher NCH concentrations, as evidenced by larger inhibition zones in all modified groups compared to the control (*p* < 0.0001).

**Conclusions:**

Incorporation of NCH into RMGIC enhanced its antibacterial activity and reduced solubility without compromising bond strength or film thickness up to 10%. However, a 20% addition adversely affected the mechanical and handling properties.

## Introduction

The clinical performance of luting cements plays a critical role in the longevity of fixed dental restorations [1]. Cement solubility, susceptibility to marginal degradation, and limited antibacterial activity contribute directly to microgap formation and biofilm accumulation, which remain major factors in the development of secondary caries at tooth-restoration interface and gradual loosening or debonding of restorations [1–4]. Long-term clinical studies reported that 8-9.5% of fixed dental prostheses (FDPs) developed secondary caries, with the risk increasing substantially in patients with poorer oral hygiene [4–7]. These limitations highlight the ongoing need for luting materials with improved stability and antibacterial potential.

Glass ionomer cements (GICs) are widely used as luting agents due to their chemical adhesion to tooth structure and continuous fluoride release. However, their moisture sensitivity during early setting and relatively low fracture resistance limit their long-term performance, particularly under high functional stresses [8–10]. Resin-modified glass ionomer cements (RMGICs) were introduced to overcome the mechanical limitations of conventional GICs by incorporating resin monomers into the formulation [9]. This hybridization enhances mechanical strength, reduces moisture sensitivity, and maintains chemical adhesion to tooth substrates, making RMGICs suitable for long-term luting of indirect restorations and orthodontic appliances [8, 10].

RMGIC establishes a strong mechanical attachment with the restoration, alongside a limited but effective chemical adhesion to the underlying tooth structure, thereby forming a fundamental interface [11]. Nevertheless, this interface remains vulnerable to microleakage, particularly in the presence of marginal discrepancies in fixed prostheses [12]. To address these limitations, various nanomaterials, chemical agents and plant extracts such as bioglass, chlorhexidine, and propolis extract have been incorporated into RMGIC to boost the material’s mechanical, physical, and antibacterial properties [8, 13–16]. The efficacy of such modifications depends on the careful optimization of additive type, concentration, and chemical compatibility, as inappropriate combinations may interfere with the setting reaction or compromise the material’s overall performance [17].

Chitosan (CH) is a biocompatible, biodegradable polysaccharide with notable antibacterial and film-forming properties, making it a promising additive for dental biomaterials [18, 19]. Nanochitosan (NCH), a nanoscale derivative of chitosan (CH), offers several advantages for dental applications due to its small particle size. The increased surface area enhances its reactivity and allows better penetration into dentinal tubules and oral biofilms. These properties lead to superior antibacterial efficacy and enhanced mechanical reinforcement of restorative materials [20]. Furthermore, NCH facilitates remineralization by chelating calcium and phosphate ions, contributing to the regeneration of demineralized dental tissues [20, 21].

Previous investigations have demonstrated that the incorporation of NCH into restorative composites, adhesives, and conventional glass ionomer systems can significantly enhance their antibacterial performance and mechanical properties, supporting its potential as a functional nanofiller for dental applications [18, 22–27]. However, limited studies have explored its incorporation into RMGI luting cements to address persistent challenges such as solubility, microleakage, and secondary caries, highlighting the need for systematic evaluation. To address this gap, the present study was designed to assess the shear bond strength, film thickness, water sorption, solubility, and antibacterial properties of an RMGIC luting cement modified with different concentrations of NCH. The null hypotheses tested were that NCH incorporation would have no significant effect on: (1) the shear bond strength to dentine, (2) the cement film thickness, (3) its water sorption and solubility, and (4) its antibacterial activity.

## Materials and methods

The materials used in the study are shown in Table 1.

**Table 1:** Materials used in the study

Materials	Composition	Manufacturer	Batch Number
Resin modified glass ionomer cement, Fuji Plus (FP, GC Corporation, Tokyo, Japan)	**Powder**: Fluoroalumino-silicate glass **Liquid**: Distilled water Polyacrylic acid (PAA) HEMA Urethane dimethacrylate (UDMA) Tartaric acid	GC Corporation, Tokyo, Japan	431,101
Chitosan nanoparticles (80–100 nm, ≥ 99.9% purity)	C₆H₁₁NO₄	Nanochemazone, Canada.	NCZ-MN-116/20
Acetic Acid	CH_3_COOH	23.3131905	CHMI-LAB NV, Belgium

### Preparation of nanochitosan solution

Chitosan nanopowder (80–100 nm, ≥ 99.9% purity; Nanochemazone, Canada) was dispersed in 0.3 N acetic acid to prepare a 0.2 mg/mL nanochitosan solution. To achieve this, 20 mg of chitosan nanoparticles was added to 100 mL of 0.3 N acetic acid, which was prepared by mixing 1.8 mL glacial acetic acid with distilled water. The mixture was magnetically stirred at room temperature until complete dispersion was achieved [18, 28, 29].

### Modification of resin modified glass ionomer liquid with nanochitosan

The liquid component of the resin-modified glass ionomer cement (RMGIC) (Fuji Plus, GC Corporation, Tokyo, Japan) was altered by adding nanochitosan (NCH) at various volume percentages: 0% (control), 5%, 10%, and 20% (v/v). In brief, NCH solutions were incorporated into the respective RMGIC liquids, then air-sealed and manually shaken gently for 5 min at room temperature to ensure mixing. The modified liquids were then stored at room temperature for 24 h prior to being combined with the RMGIC powder. The pH of all the modified liquids remained consistent, measuring approximately 1.0. Finally, each modified liquid and the unmodified control were mixed with the RMGIC powder following the manufacturer’s guidelines.

### Shear bond strength (SBS) test

#### Teeth selection, preparation and bonding to dentin

Following ethical approval from the Ethics Committee of the Faculty of Dentistry, Mansoura University, Egypt (Approval No. R.25.07.08, approved on July 8, 2025), freshly extracted sound human mandibular first molars, extracted for periodontal reasons, were collected from patients aged 35–60 years. Teeth presenting with carious lesions, visible cracks, fractures, restorations, or developmental anomalies were excluded to ensure sample integrity. After removal of residual debris, specimens were kept in 0.1% thymol solution for 30 days to inhibit microbial growth, then transferred to distilled water and maintained at 4 °C until further use. A total of 40 teeth (*N* = 40, *n* = 10 per group) fulfilling the inclusion criteria were selected. Sample size determination was performed using G*Power software (Düsseldorf, Germany), with parameters set at α = 0.01, power = 95%, and an anticipated effect size of 0.25, confirming sufficient statistical power for the study. The teeth were mounted in acrylic resin blocks, ensuring that the occlusal surfaces were oriented parallel to the base. The coronal portions were sectioned using a water-cooled diamond saw (Isomet 4000: Buehler Ltd., IL-USA) to expose mid-coronal dentine under controlled conditions and to prevent thermal damage. To standardize the smear layer, the exposed dentine surfaces were polished using 600-grit silicon carbide papers under continuous water irrigation for 60 s. The cement was mixed as per the manufacturer’s instructions. RMGIC was applied using a cylindrical silicone split mould (height: 2 mm; diameter: 4 mm). With the silicon mould set on the dentine surface, RMGIC was inserted into the mold and left to set. The mold was slightly overfilled and pressed with a glass slab, and the excess was removed. The specimens were retrieved after 10 min by separating the silicone molds exerting minimal stress, and were stored at 37 °C and 100% humidity for 24 h to ensure complete maturation [30].

#### Thermocycling, shear bond strength testing and fracture analysis

All specimens were subjected to thermocycling for 10,000 cycles between 5 °C and 55 °C, with a dwell time of 30 s in each bath and a transfer time of 5 s, to simulate approximately one year of clinical function under oral thermal conditions [31]. The specimens were subsequently mounted on the jig of a universal testing machine (Model Instron UTM 5582), where shear force was applied at a crosshead speed of 0.5 mm/min until failure occurred. The SBS was determined by dividing the load at failure by the bonded surface area [30]. A stereomicroscope (Olympus SZ1145; Olympus Optical Co. Ltd., Japan), set to 20× magnification, was used to examine the fractured surfaces after SBS testing through visual examination to categorize the failure mode. Failures in each group were categorized into the following categories: “dentine-cement interface adhesive failure,” “cement cohesive failure,” “dentine cohesive failure,” or “mixed failure” [32].

### Film thickness measurement

**Fig. 1:**
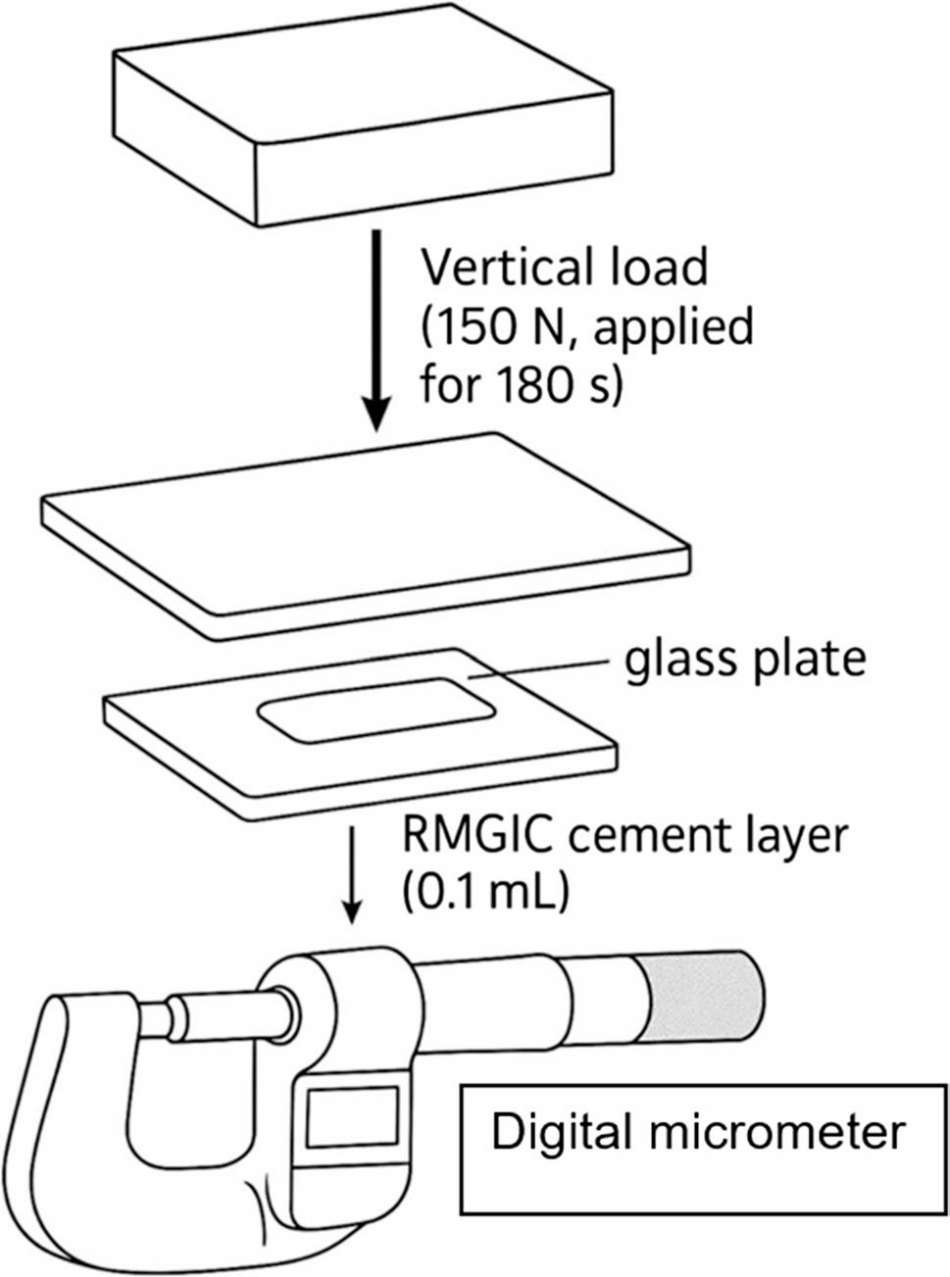
Schematic representation of film thickness measurements setup

Film thickness was assessed according to the American National Standards Institute/American Dental Association (ANSI/ADA) Specification No. 8 for zinc phosphate cement and the International Organization for Standardization (ISO) Specification 9917-1 for water-based cements. Two flat glass plates with a contact area of 200 mm² were placed in direct contact, and their combined thickness was precisely measured using a digital micrometer (Mitutoyo 342 − 251; Sakado, Japan). Three measurements were taken, and the average value was recorded as the baseline thickness, designated as measurement (A). Each RMGIC luting agent was first prepared according to the manufacturer’s recommended protocol, and a small volume (0.1 mL) was then accurately dispensed using a 1 mL graduated insulin syringe onto one of the glass plates. Ten seconds after the initiation of mixing, a second glass plate was positioned over the first, and a vertical load of 150 N was applied for 180 s under controlled conditions using a universal testing machine, in accordance with ISO 4049:2019 (Fig. 1). Following load application, the combined thickness of the plates with the cement layer was measured using the same digital micrometer technique and recorded as measurement (B). The film thickness of the RMGIC was determined by subtracting the initial glass plate thickness (A) from the final measurement (B). Ten specimens were prepared for each group, and the mean film thickness ± standard deviation (SD) was calculated [33].

### Water sorption and solubility test

Disc-shaped specimens were prepared using Teflon moulds with an internal diameter of 15 ± 0.1 mm and a thickness of 1 ± 0.1 mm. Each mold was placed on a glass plate covered with a Mylar strip. The restorative materials were mixed and handled in accordance with the manufacturers’ instructions, then slightly overfilled into the moulds. A second Mylar strip was placed over the material, followed by another glass plate. Gentle pressure was applied to ensure uniform thickness and eliminate voids, while excess material was carefully removed. Specimens were demoulded 10 min after mixing and subsequently stored in a dry environment at room temperature for 24 h to allow for complete setting. Each specimen was carefully examined visually against a light source to identify any internal porosities. The edges were manually smoothed using dry 600-grit silicon carbide abrasive paper (Buehler, IL, USA) to remove any irregularities. Residual debris was cleared away using the dental unit’s air syringe. Specimens showing porosities or surface defects were discarded from the study.

Following surface preparation, specimens were placed in a vacuum desiccator maintained at 37 ± 1 °C for 22 h, then transferred to a second desiccator at 25 ± 1 °C for an additional 2 h. Subsequently, each specimen was weighed using a calibrated digital analytical balance (Sartorius, Germany). This drying-weighing cycle was repeated until a constant mass was achieved, which was recorded as the initial dry weight (*W₀*). The specimens were then immersed in artificial saliva (pH 7) and stored in an incubator at 37 ± 1 °C for 28 days to simulate intraoral aging. The artificial saliva was prepared according to a previously published formulation and had the following composition (per 1000 mL): potassium chloride (0.96 g), sodium chloride (0.67 g), magnesium chloride (0.04 g), potassium phosphate (0.27 g), calcium chloride (0.12 g), methyl paraben (0.01 g), propyl paraben (0.10 g), methyl p-hydroxybenzoate (8.0 g), sorbitol (24 g), and distilled water (1000 mL) [34].

At the end of the storage period, specimens were gently dried with absorbent paper to remove surface moisture and reweighed to determine the post-immersion weight (*W₁*). Using the same protocol applied for obtaining *W₀*, the specimens were reconditioned in the desiccators until a constant weight was achieved again, which was recorded as (*W₂*). Water sorption (*W*_*sp*_) and solubility (*W*_*sl*_) values, expressed in µg/mm³, were calculated for each specimen using the following equations [35]:

$$W_{sp}=\left(W_1-W_2\right)/V$$

$$W_{sl}=\left(W_0-W_2\right)/V$$

where *W*_*0*_ is the weight of specimen (µg) before immersion in artificial saliva, *W*_*1*_ is the weight of specimen (µg) after immersion in artificial saliva, *W*_*2*_ is the dehydrated weight and *V*-volume of specimens (mm^3^). The volume (V) of each specimen, in mm³, was calculated using the average thickness and diameter according to the following formula:

$$V=\pi\times r^2\times h$$

where r is the mean sample radius in millimeters and h is the mean sample thickness in mm.

### In vitro antibacterial activity

Ten disc-shaped specimens (10 mm diameter × 2 mm height) were fabricated for each group using a split Teflon mould. Antibacterial activity was assessed against *Streptococcus mutans* (ATCC 25175) using the agar disc diffusion method. The bacterial strain was subcultured from stock onto Brain Heart Infusion (BHI) broth and incubated at 37 °C for 24 h to achieve active bacterial growth. Bacterial growth was assessed by observing turbidity in the broth following incubation, which was evaluated visually by comparing the inoculated tube to turbidity standards against a black background. Fresh *Streptococcus mutans* cultures were then inoculated onto BHI agar plates and uniformly spread using sterile cotton swabs to ensure even distribution across the surface. To ensure asepsis, the prepared RMGIC specimens were sterilized by heating in a hot air oven at 160 °C for one hour. After cooling, the specimens were aseptically placed onto BHI agar plates using sterile forceps and incubated at 37 °C for 48 h. The diameters of the resulting bacterial inhibition zones surrounding each specimen were measured at three separate locations using a digital calliper (Mitutoyo, Tokyo, Japan), and the mean value was recorded. The inhibition zone size was determined by taking the average of the three measured diameters and deducting the 10 mm diameter of the specimen from this average [36, 37].

### Statistical analysis

Prior to conducting the tests, Shapiro-Wilk test was used to verify the normality of distribution. Data was tabularized to be ready for statistical analysis, that was executed utilizing SPSS software, version 26. Means as well as standard deviations for each test were determined. Data were analyzed with one-way ANOVA succeeded by Tukey’s post hoc test (α = 0.05).

## Results

### Shear bond strength

**Fig. 2:**
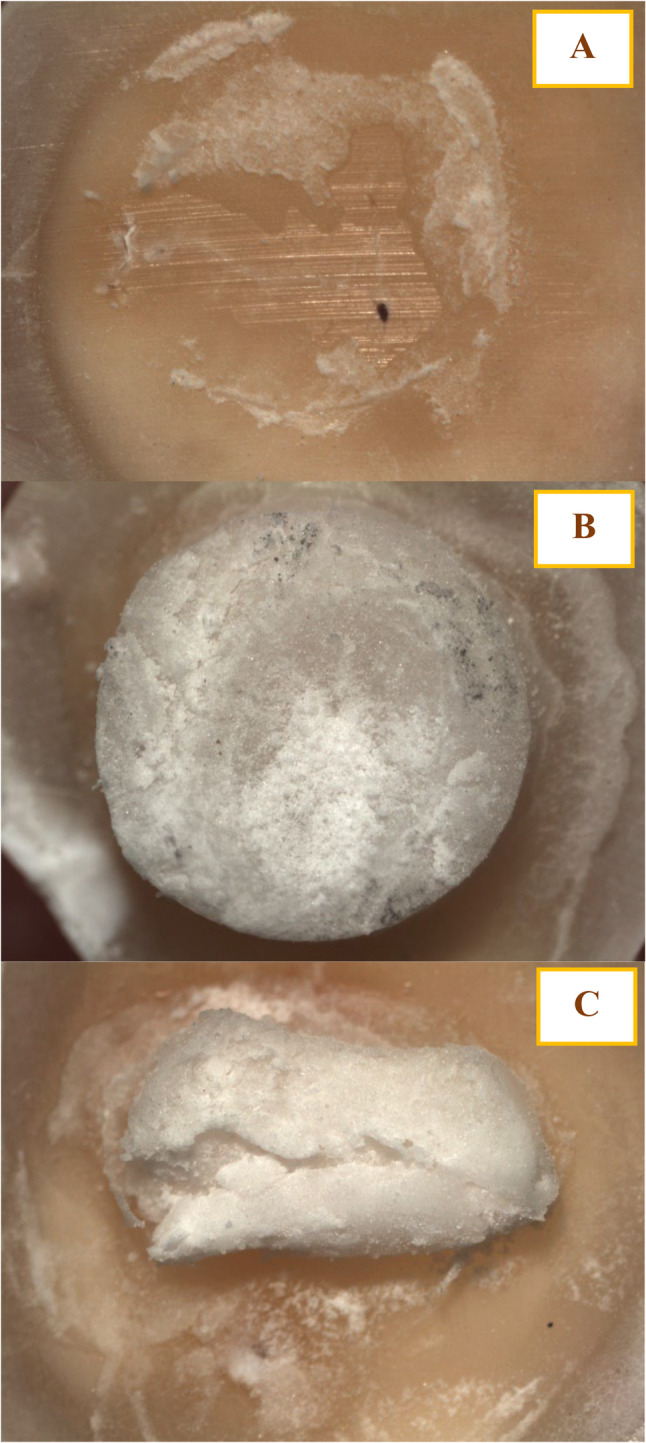
Representative bond failure modes observed after shear bond strength testing: (**A**) Adhesive failure, (**B**) Cohesive cement failure, and (**C**) Mixed failure

**Fig. 3:**
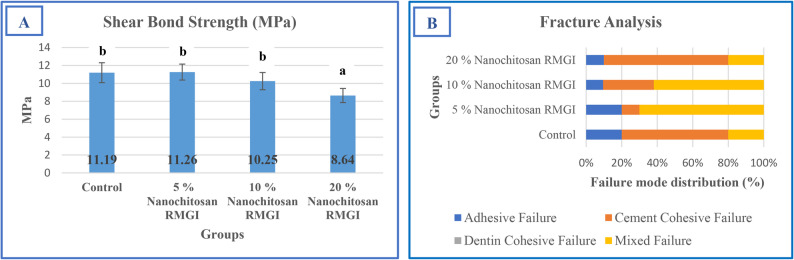
**A** Shear bond strength of control and nanochitosan-modified RMGIC groups (mean ± SD, *n* = 10). Bars with different letters indicate statistically significant differences between groups (*p* ≤ 0.05, Tukey’s post hoc test). **B** The inset bar chart shows the percentage distribution of failure modes among fractured specimens

A significant reduction in shear bond strength was observed in the 20% nanochitosan group (8.64 ± 0.79 MPa) compared to the control, 5%, and 10% groups (*p* ≤ 0.05). In contrast, no significant differences were detected among the control (11.19 ± 1.11 MPa), 5% (11.26 ± 0.89 MPa), and 10% (10.25 ± 0.96 MPa) groups (*p* > 0.05), suggesting that the adverse effect on bond strength becomes evident only at higher nanochitosan concentrations. Failure mode analysis revealed predominantly cement cohesive failure in the control group, a shift toward mixed failure in the 5% and 10% groups and primarily cement cohesive failure (70%) in the 20% group, indicating that the decrease in bond strength was mainly due to internal structural compromise rather than interfacial debonding. Representative examples of the observed adhesive, cohesive, and mixed failure patterns are presented in Fig. 2. The shear bond strength and failure mode distribution are summarized in Fig. 3.

### Film thickness

**Fig. 4:**
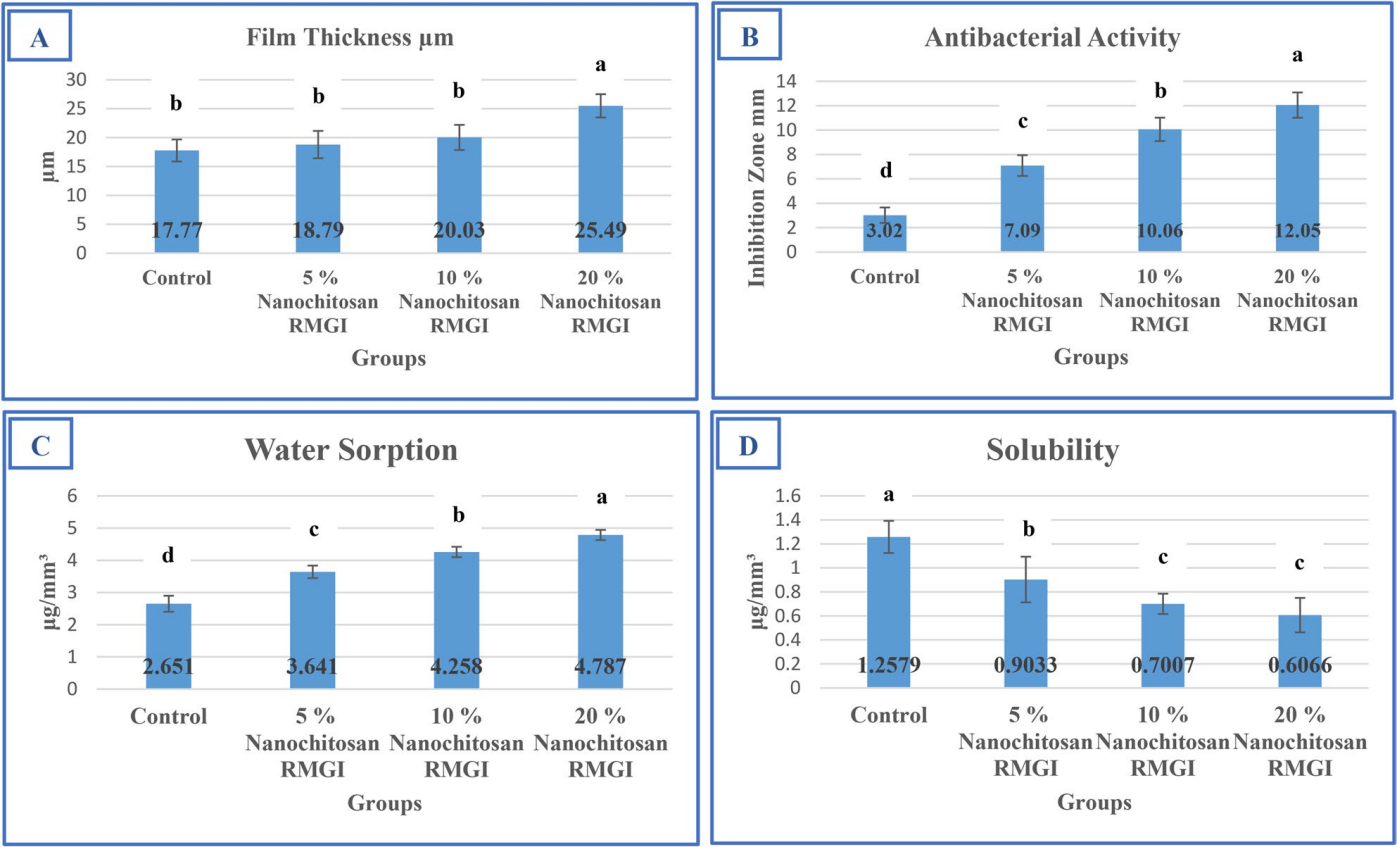
Effect of nanochitosan incorporation into resin-modified glass ionomer cement (RMGIC) on (**A**) film thickness, (**B**) antibacterial activity (inhibition zone diameter), (**C**) water sorption, and (**D**) solubility. Data are presented as mean ± SD (*n* = 10). Different letters above the bars indicate statistically significant differences among groups within each test (*p* ≤ 0.05, one-way ANOVA followed by Tukey’s post hoc test)

A statistically significant increase in film thickness was evident only in the 20% nanochitosan group (25.49 ± 2.01 μm) compared to all other groups (*p* ≤ 0.05). No significant differences were found among the control (17.77 ± 1.90 μm), 5% (18.79 ± 2.36 μm), and 10% (20.03 ± 2.17 μm) groups (*p* > 0.05) (Fig. 4).

### Water sorption

Water sorption increased progressively with higher nanochitosan loading (*p* < 0.0001). The 20% group demonstrated the highest mean value (4.79 ± 0.16 µg/mm³), while the control group had the lowest (2.65 ± 0.25 µg/mm³). All intergroup differences were statistically significant (*p* ≤ 0.05) (Fig. 4).

### Water solubility

Solubility values decreased significantly with increasing nanochitosan content (*p* < 0.0001). The 20% group exhibited the lowest solubility (0.61 ± 0.14 **µ**g/mm³), while the control group had the highest (1.26 ± 0.13 µg/mm³). All chitosan-modified groups showed significantly lower values compared to the control (*p* ≤ 0.05) (Fig. 4).

### Antibacterial activity

**Fig. 5:**
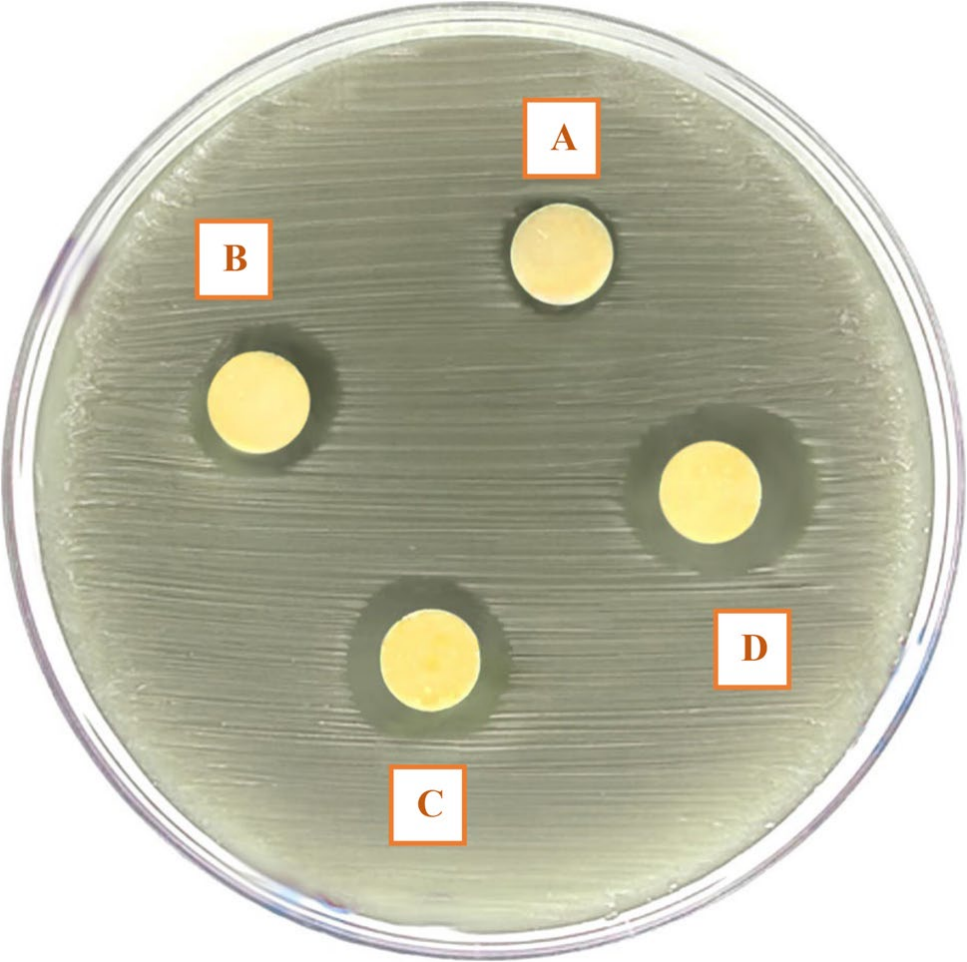
Representative inhibition zone images illustrating antibacterial activity of (**A**) Control, (**B**) 5% nanochitosan-modified RMGIC, (**C**) 10% nanochitosan-modified RMGIC, and (**D**) 20% nanochitosan-modified RMGIC

Antibacterial effectiveness, assessed by inhibition zone diameter, increased significantly with higher concentrations of nanochitosan (*p* < 0.0001). The 20% group showed the largest inhibition zone (12.05 ± 1.04 mm), followed by 10% (10.06 ± 0.96 mm), 5% (7.09 ± 0.85 mm), and control (3.02 ± 0.64 mm), with all differences statistically significant (Fig. 4). Representative images of the inhibition zones are shown in Fig. 5.

## Discussion

This study investigated the effects of incorporating nanochitosan (NCH) into the commercial polyacrylic acid (PAA) liquid of resin-modified glass ionomer (RMGI) luting cement through four key evaluations: shear bond strength to dentine, film thickness, water sorption and solubility, and antibacterial activity against *Streptococcus mutans*. The results indicated that the impact of NCH varied across the tested properties. While lower concentrations of NCH did not significantly affect the shear bond strength, higher concentrations led to a noticeable decrease, resulting in partial rejection of the first null hypothesis. A significant increase in film thickness was observed only at higher concentrations of NCH, resulting in partial rejection of the corresponding null hypothesis. In contrast, water sorption increased and solubility decreased consistently across all NCH concentrations, supporting full rejection of the related null hypothesis. Additionally, the incorporation of NCH significantly enhanced the antibacterial activity of RMGIC in a concentration-dependent manner, justifying the rejection of the fourth null hypothesis. Overall, these findings suggest that the incorporation of NCH, particularly at lower concentrations, can positively influence the antimicrobial and physicochemical behaviour of RMGIC, without compromising its adhesive performance.

At lower concentrations, the incorporation of NCH did not significantly influence shear bond strength to dentine. This finding suggests that adhesive mechanism, primarily based on ionic interactions between the carboxyl groups of polyacrylic acid and calcium ions in hydroxyapatite, remains functionally preserved [32]. At these lower concentrations, NCH particles are assumed to be uniformly dispersed within the cement matrix and do not interfere with the diffusion or chelation mechanisms necessary for effective chemical bonding to dentine. Additionally, the hydrogen-bonding potential of chitosan’s amino and hydroxyl groups may support cohesive interactions within the cement matrix, preserving its mechanical and adhesive integrity [32, 38].

Furthermore, micromechanical bonding to tooth structure appears unaffected at low NCH concentrations. This could be attributed to the maintenance of the resin phase properties, particularly those of hydroxyethyl methacrylate (HEMA), which are responsible for penetrating dentinal micro-irregularities and facilitating micromechanical interlocking [39]. At these concentrations, NCH does not appear to significantly alter the rheological behaviour, film thickness, or viscosity of the RMGIC, parameters that are critical for optimal flow and adaptation to the tooth substrate. Therefore, the dual bonding mechanism of RMGIC, involving both ionic interaction with hydroxyapatite and micromechanical retention, remains functionally effective [39].

Failure mode analysis revealed a shift from cohesive cement failure in the unmodified group to predominantly mixed failure in the 5% and 10% NCH-modified groups. This change may be attributed to the mechanical reinforcement effect of NCH, which likely enhanced the structural integrity of the RMGIC matrix, thereby reducing internal failure within the cement [32].

At higher concentrations, NCH may exhibit self-association, leading to intermolecular interactions between NCH particles rather than effective bonding with the RMGIC matrix. This may result in agglomeration or phase separation, thereby limiting NCH integration and contributing to the observed reduction in shear bond strength to dentine [40]. Furthermore, higher concentrations of NCH may increase viscosity and impair flow, potentially limiting resin infiltration and compromising micromechanical bonding, as indicated in the film thickness results. This adverse effect of high NCH content on the reinforcement of RMGIC could explain the predominance of cement cohesive failure observed in the 20% NCH-modified GIC specimens. Previous literature has shown that excessive amounts of chitosan may negatively affect mechanical performance due to molecular aggregation and interference with the setting reaction of GICs. However, at the controlled concentrations used in this study, no such detrimental effects were observed on bond strength, aligning with the findings of Ibrahim et al. who reported stable adhesion of chitosan-modified GIC to dentine [32].

Film thickness is a critical parameter for luting cements, as excessive thickness can interfere with the complete seating of indirect restorations and affect marginal adaptation [41–43]. In the present study, NCH incorporation into the polyacrylic acid (PAA) liquid of RMGIC exhibited a concentration-dependent effect on film thickness. At lower concentrations, NCH did not significantly alter the film thickness, whereas higher concentrations resulted in a measurable and statistically significant increase. Despite this, the measured film thickness remained within the clinically acceptable limit of 25 μm [41], suggesting that the modified cement could still be suitable for permanent luting applications.

This outcome may be attributed to the physical and rheological properties of NCH. As a high molecular weight cationic polysaccharide, NCH tends to increase the viscosity of the liquid phase when incorporated at higher concentrations [32, 38]. The increased viscosity reduces the flowability of the mixed cement, thereby increasing its resistance to pressure during seating and resulting in thicker cement films [44]. In contrast, at lower concentrations, NCH is likely more uniformly dispersed, resulting in minimal impact on the flow properties of the cement [18]. These findings support the suitability of NCH-modified RMGIC in permanent luting applications and highlight the importance of optimizing NCH concentration to maintain proper handling and adaptation.

Water sorption and solubility are essential parameters that influence the long-term durability and dimensional stability of luting cements [45]. In this study, the incorporation of NCH resulted in a significant increase in water sorption and a simultaneous reduction in solubility, particularly at higher NCH concentrations. The observed increase in water sorption could be attributed to the inherently hydrophilic nature of CH, which possesses free amino (-NH₂) and hydroxyl (-OH) groups capable of forming hydrogen bonds with water molecules [46]. At the nanoscale, the large surface area of NCH particles enhances their interaction with the aqueous environment, facilitating water uptake into the cement matrix [47]. Additionally, NCH contributes to the formation of a porous polymeric network with nanoscale voids capable of retaining water molecules, both as free water within the pores and as water bound to its hydrophilic functional groups [48]. Clinically, increased water sorption may result in slight dimensional changes or swelling over time, potentially affecting marginal adaptation or seating of restorations [49]. While the concurrent reduction in solubility may mitigate these effects and indicate improved material stability and potential longevity, the possible adverse impact should be acknowledged, particularly as the modified RMGIC has not yet been tested under clinical conditions.

These findings are in line with previous studies showing that the modification of GICs and RMGICs with biopolymers increases water affinity due to enhanced surface polarity [50, 51]. In contrast, the reduction in solubility observed with NCH incorporation may be explained by stronger intermolecular interactions and potential crosslinking between the amino groups of NCH and the carboxyl groups of polyacrylic acid. These interactions likely result in the formation of a more cohesive and stabilized matrix, which exhibits increased resistance to dissolution in aqueous environments [38]. Additionally, CH has been reported to interact with hydroxyl groups on glass particles and to carboxylic groups in PAA, primarily through hydrogen bonding. The resulting chitosan-PAA network surrounding the inorganic particles is thought to reduce interfacial tension among the glass ionomer components, enhancing mechanical performance and contributing to reduced solubility by limiting the diffusion of unreacted and soluble species [18, 38]. Furthermore, unreacted NCH particles may act as inert fillers within the RMGIC matrix, reducing porosity and limiting the diffusion of soluble components, which could contribute to improved structural integrity [18]. These findings suggest that while NCH-modified RMGIC absorbs more moisture, its resistance to disintegration in water improves, potentially enhancing its clinical longevity. However, careful control of NCH concentration is important to balance water sorption and maintain dimensional stability, particularly in moisture-sensitive clinical environments such as subgingival margins.

The antibacterial activity of RMGIC was significantly enhanced by the incorporation of NCH, with the effect being concentration-dependent. The observed reduction in *Streptococcus mutans* growth can be attributed to the known antimicrobial properties of chitosan. Under acidic conditions, chitosan’s amino groups become protonated, allowing electrostatic interactions with negatively charged bacterial cell membranes. This interaction compromises membrane integrity, leading to leakage of intracellular contents and eventual bacterial cell death [52]. Moreover, nanosized chitosan particles may penetrate the bacterial cell wall and interact with DNA, disrupting replication and protein synthesis [53]. These results are consistent with previous studies demonstrating that CH-modified GICs exhibit enhanced antibacterial properties, increased fluoride ion release, and improved resistance to bacterial colonization [32, 38, 54]. Notably, in this study, the enhanced antibacterial effect was achieved without compromising adhesion at lower concentrations of NCH. This highlights the potential utility of NCH as a biofunctional additive to enhance the biological performance of RMGICs.

Overall, lower NCH concentrations (5–10%) preserved shear bond strength, maintained clinically acceptable film thickness, and enhanced antibacterial activity, whereas higher concentrations (20%) caused reduced bond strength and increased film thickness. This highlights the importance of optimizing NCH concentration to achieve a balance between mechanical performance, handling properties, and antimicrobial efficacy for potential clinical applications.

## Conclusions

Considering the limitations of this in vitro study, it can be concluded that the incorporation of NCH into the liquid component of RMGI luting cement enhances antibacterial efficacy and reduces solubility. At lower concentrations (5–10%), these enhancements were achieved without negatively impacting shear bond strength or compromising clinically acceptable film thickness. However, higher concentrations (20%) were associated with reduced bond strength and increased film thickness, indicating potential adverse effects. These outcomes support the potential use of lower NCH concentrations as functional additives to enhance the physical, mechanical, and antibacterial performance of RMGICs.

### Limitations

This study was conducted under controlled in vitro conditions, which do not fully mimic the complex and dynamic nature of the oral environment, including pH fluctuations, enzymatic activity, and mechanical stresses. The long-term durability of the antibacterial effect and the material’s physical properties over time, particularly in relation to aging and degradation processes, were not evaluated. Furthermore, antibacterial testing was limited to *Streptococcus mutans*, without assessing the material’s effectiveness against other cariogenic or biofilm-associated microorganisms. Therefore, additional in vivo studies are essential to validate the clinical applicability, biocompatibility, and long-term performance of NCH-modified RMGICs.

## Data Availability

The datasets generated and/or analyzed during the current study are available from the first author “Mostafa A. Abdelshafi” upon request.
